# The Evolution of the Modern Hospital

**Published:** 1915-04-17

**Authors:** B. Burford Rawlings


### THE EVOLUTION OF THE MODERN HOSPITAL.*

### VII.—The Problem of the Unpaid Staff.

By B. BURFORD RAWLINGS.

When we regard a hospital as purely philanthropic and therapeutical in its action, and overlook that it is also a school, we most nearly approach the point of view occupied by an overwhelming majority of the patients. To them a hospital is a place of healing; it exists to minister to their wants, and they are in no way disposed to acquiesce in the notion that they have entered the hospital for anybody's benefit but their own. The poorer and more ignorant patients are either suspicious of everything or they display a trustful selfishness, which enables them to enjoy the comfortable belief that all the complex machinery of the hospital has been erected and set in motion for the benefit of themselves and their class, while in the multitudinous questionings of the residents, the solicitous interest of the clerks and students, the copious records of their symptoms and progress, and the other details of the day's routine, they see accumulating evidence of the serious efforts called forth by the importance of their individual claim to cure.

To the patients not hypnotised by the unwonted comfort of a hospital ward, other reflections present themselves. In some instances they become morbidly sensitive to the suspicion that they have been admitted less with a view to their own advantage than to serve as object-lessons; and once they have persuaded themselves of this, the temperature chart, the weighing machine, and the camera, so far from indicating beneficent efforts to mend their condition, are but evidences of a wearisome iteration of demands that their bodies should yield fruits to research.

The objection of the doctor to contributions by patients, which, cultivated systematically, would lend valuable help to hospital finance, arises partly from the fact that the mental attitude of contributing patients towards himself differs essentially from that of the poorer inmates. They take a more independent view of their position. They are less affected by the glamour of his presence and pretensions, they do not yield themselves so readily to demonstration, and hence limits are set to their clinical value.

But in one respect at least the position of all hospital patients is the same. Nobody is free to question anything done or omitted to be done by a medical officer without danger of grave consequences; and whatever liberty of language or demeanour a patient is impelled to by impatience, temper, or other infirmity of sick folk, he must be careful that it is never displayed in the hearing or presence of "the doctor." A patient who thinks himself aggrieved in anything affecting his medical treatment is in this dilemma: he cannot afford to be proved in the wrong (as he almost certainly will be), still less can he safely venture to show himself in the right. In either case disaster to his own prospects will follow. When a patient pulled out a quantity of a nurse's hair, scratched her face, tore her clothing, and was with difficulty restrained from other violence, and became a veritable terror in the ward, the medical residents, backed by the visiting physician, called in question the nurse's management and refused to discharge an interesting case. When the same woman gave a blunt answer to a question put by a house physician she was instantly discharged. It is not enough that medical considerations should take precedence of all others; few would object to that; they must displace them; and patients soon learn that the safest course is to accept the general view of the doctor's autocracy.

Still, the fact remains that the sick do not of their own free will surrender themselves unconditionally into the hands of the staff, and any hospital which demanded of them this renunciation as a prelude to admission would soon meet with a dearth of patients. Some people might question this, but how long would the well-earned confidence of the private patient in his doctor survive the introduction of the clinical factor into the ministrations afforded him? Let him once get to know that there are actuating causes at work other than the desire for his relief, and other interests to serve possibly consistent with, but certainly distinct from, his own, and he would soon begin to suspect that upon occasion the other interests would be given preference to his detriment. To say that everybody who makes use of a doctor incurs a debt to the hospitals is but to urge the obvious. Private patients, usually without a thought of obligation, benefit by the fact that one function of hospitals is to teach. The hospital patient it is who may be called upon to make payment in kind.

Of late years economy in hospital administration has been much insisted upon, and suggestions more or less useful have been made in plenty; but as nobody seems to have attempted to deal with extravagance at its source, the results have been disappointing. Transcending all other influences upon expenditure is the disposition of members of the staff, and, according to their attitude, the domestic expenses of any hospital may range from profusion to parsimony. Mostly, the staff are indifferent to the difficulties of finance, and unless the relations of lay and medical government happen to be unusually harmonious, representations made with a view to reduce expenditure would be coldly received. The medical mind is admittedly arbitrary; it is impatient of suggestion, and as scarcely an item of domestic expenditure is independent of medical orders, lay efforts to induce economy have little chance of success. Illuminating stories might be told of thwarted attempts in this direction, which have brought about results the precise opposite of those aimed at. In hospitals, much which private patients dispense with is accounted necessary,

* Previous articles: February 20, March 6, 13, 20, 27; and April 3, 10.

while costly novelties in drugs, instruments, and appliances are requisitioned, sometimes only to be discarded after trial. Under existing conditions, the dieting of a patient is left to the discretion of a house surgeon or house physician, who, with youthful indifference to ways and means, possesses powers of expenditure, virtually uncontrolled, to be appreciated only by those who have witnessed their exercise and have shouldered the burden of hospital poverty. Laymen cannot of themselves do anything in these matters, because by way of resistance it is always asserted that the medical interests of patients are involved. Almost as impossible is it to prevent waste of lighting and fuel in consultation rooms, laboratories, and the private rooms of residents, who habitually omit to recognise the little duties self-imposed by careful householders, and have empty apartments heated and illuminated for hours together. To appeal to the members of the staff is as useless as to reason with the resident, who wraps himself in professional exclusiveness and stands fast upon his medical prerogative.

In the hospital possessing the customary equipment of a non-resident lay authority and a number of medical residents, the severance between the lay and medical worker is almost complete. Each takes his view from his own standpoint. The layman thinks that patients are admitted to the care of the hospital, while contrariwise the members of the staff and the residents regard them as severally apportioned among themselves. The not uncommon notion that the collective attention and experience of the whole staff is brought to bear upon individual patients is, of course, only ludicrous. Nevertheless the distinction between being committed to the care of a single unit of the medical body attached to the hospital and being a patient of the hospital itself is neither so subtle nor so unimportant as at first it may appear.

The hospital is a living organism, and something is wanted to satisfy both the subscriber and the patient that it is not invertebrate, and that all its functions are under control. The medical work of the wards, especially that part of it concerned with the utilisation of patients for teaching purposes, needs to be subject to competent scrutiny, coupled with power to intervene at discretion. At present an interesting case, no matter what discomfort is suffered by the patient, may be exhibited and talked about several times in a day as medical visitors or friends of residents drop in. Manifestly a power of control in these matters could not be exercised by laymen nor by any sort of committee. The authority must be personal and always at hand, while a conjunction of qualities is needed rarely united, the possession of medical knowledge combined with a full aceptance of the fact that a hospital is first benevolent, then educational, which is precisely what the untempered professional mind is incapable of comprehending.

If the assertion that the problem of finance governs the situation is a truism, to say that the difficulties will be best overcome by taking a step involving a large addition to expenditure may partake of paradox. Yet nothing will more certainly bring the relations of staff, school, and hospital into harmony and assist finance than the application of principles which obtain unquestioned adoption in all other affairs of life. Sentimentally considered, unpaid service appears to consist admirably with philanthropic effort, but if the use of a commercialism is permissible, it is not business, and hospital administration is serious business. Direct payment of the staff is essential to the salvation of the hospitals as voluntary institutions, because it alone can make the governing body master in its own house. Payment of the visiting staff would need to be accompanied by a revision of the conditions of resident service, involving better accommodation and a substantial increase of remuneration. Resident appointments should be disconnected from the school to the extent that they should be no longer appropriated by it as prizes for successful students. A great deal more than mere scholarship is needed to make a satisfactory resident. Graduates, fully qualified in years and professional attainments, prepared to hold office during a minimum term of two years, should be substituted for the house physicians and house surgeons as we know them. Men of the age and professional standing of the present holders of office should be given non-resident subordinate posts, with salary, tenable for six or twelve months, and should perform their duties under the supervision of the residents, who, as will be presently suggested, should be subject to a medical chief.

It has been said that the distinguished physicians and surgeons upon the staffs would not consent to paid service. The difficulty, if it arose, would prove no more than temporary, and with a better conception of the demands of hospital duty the proposition that honorary service is out of place would soon cease to offend. At more than one leading hospital small and quite insufficient payment has always obtained, and in other instances fees nominally to cover disbursements are not unknown. These payments have not proved unwelcome to the recipients, and it is certain that many young and promising men have been hindered in their careers because they could not afford to work without payment. Unpaid office has attractions for those who desire its freedom, but a claim for freedom to perform at will, and only to the extent agreeable, duties which affect the well-being of other people, is indefensible. Obviously, the relation of a governing body to an unpaid staff is one of difficulty. Boards of governors may be termed supreme, but they are seldom in doubt concerning their limitations. Their action must always stop short of the heart of things; it must be directed to what is merely accessory and subordinate. Newly elected members are sometimes surprised at a discovery of the impotence they suffer in their relations with the staff, and not seldom arrive at knowledge by way of humiliation. Such was the experience of a certain field officer of distinction called upon one day to preside over the house committee of a well-known general hospital. When this gentleman learned that a member of the staff, not for the first time, was absent from duty without notice, he dispatched a telegram to the truant requiring him to attend at once, and to report himself to the next house committee. Of course the physician did neither of these things. Anybody familiar with hospital ways will understand the amazement the incident must have caused him, and as his colleagues rallied at once, the difficulty had to be adjusted by the committee with some surrender of dignity. When we examine this occurrence we realise the correctness of the chairman's act as an abstract proceeding, and, looked at in the light of hospital custom, its extreme unwisdom. The only result was to expose afresh the futility of attempting an exercise of authority, however legal and reasonable, over the members of an honorary staff, and, as matters are, a lay board cannot find a way of dealing promptly even with salaried medical officers, backed up, as almost invariably they are, by their unpaid seniors.

